# A novel non-viral *PDCD1* site-integrated CAR design: killing 2 birds with 1 stone

**DOI:** 10.1097/BS9.0000000000000135

**Published:** 2022-10-10

**Authors:** Yuanbin Cui, Yunlin Huang, Le Qin, Peng Li

**Affiliations:** a China-New Zealand Joint Laboratory of Biomedicine and Health, State Key Laboratory of Respiratory Disease, Guangdong Provincial Key Laboratory of Stem Cell and Regenerative Medicine, Key Laboratory of Stem Cell and Regenerative Medicine, Guangzhou Institutes of Biomedicine and Health, Chinese Academy of Sciences, Guangzhou, China; b Centre for Regenerative Medicine and Health, Hong Kong Institute of Science & Innovation, Chinese Academy of Sciences, Hong Kong SAR, China

**Figure 1.**
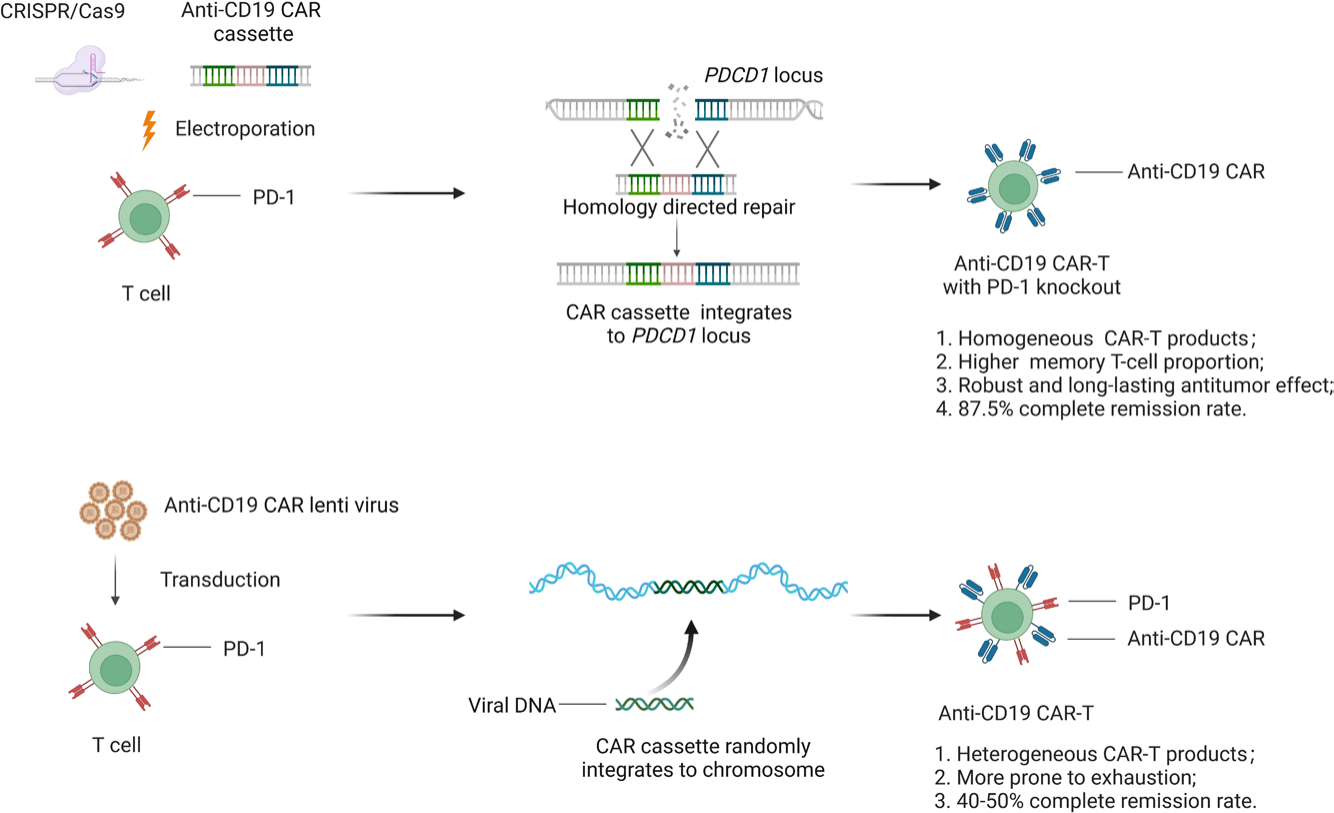
Schematic representation of the viral CAR-T-cell and non-viral *PDCD1* site-integrated CAR-T-cells production. (A) Lentiviruses deliver CAR cassette by randomly integrating it to T cell chromosome. Immune checkpoint molecule PD-1 is still expressed on CAR-T-cell surface and make it more prone to exhaustion. (B) By using CRISPR/Cas9 technology, CAR cassette specifically inserts into the *PDCD1* locus in non-viral *PDCD1* site-integrated CAR-T-cell. By knocking out the *PDCD1*, non-viral *PDCD1* site-integrated CAR-T-cell product has higher memory T cell proportion with long lasting antitumor effect. CAR-T-cell = chimeric antigen receptor T-cell.

Although chimeric antigen receptor T-cell (CAR-T-cell) therapy has shown excellent efficacy against refractory/relapsed B-cell lymphoma, B-cell acute lymphoblastic leukemia and multiple myeloma,^1,2^ the complete response rate of patients with refractory/relapsed B-cell lymphoma receiving conventional CAR-T-cell therapy is approximately 40% to 50%.^3–5^ There are 3 drawbacks to viral CAR-T-cell therapy. First, random integration of the CAR cassette and lentivirus replication are potential risks for viral CAR-T-cell therapy (**Fig. 1A**).^6–8^ Second, the expression levels of CAR vary in different CAR-T-cells, depending on their integration sites, which may affect the efficacy and toxicity of this therapy. In addition, the complex manufacturing process and long preparation time of viral-derived CAR-T-cells needs improvement. To overcome these hurdles, several teams have used CRISPR−Cas9 to integrate the CAR cassette into specific genome loci, including TRAC and AAVS loci.^9,10^

The programmed cell death receptor-1 (PD-1)/programmed cell death-ligand 1 (PD-L1) axis is considered one of the most important immunosuppressive signaling pathways in T-cells. Undoubtedly, the anti-tumor efficacy of CAR-T-cells is also hindered by the PD-1/PD-L1 axis. To date, various types of immune checkpoint inhibitors targeting the PD-1/PD-L1 axis have been approved for the treatment of a number of tumors.^11,12^ However, owing to potential safety concerns and the difficulty of co-localization of CAR-T-cells and PD-1/PD-L1 inhibitors,^13^ the combination of CAR-T therapy and PD-1 inhibition needs to be further optimized.^13–15^ Previous studies have demonstrated that disrupting PD-1 in CAR-T-cells by CRISPR−Cas9^16^ or converting immunosuppressive PD-L1 signaling into stimulatory signaling by chimeric switch receptor T (CSR-T) cells can augment the anti-tumor effects of CAR-T-cells.^17,18^

Recently, the teams of Mingyao Liu and He Huang (et al) developed a two-in-one approach to generate non-viral CAR-T-cells by inserting an anti-CD19 CAR cassette into the specific locus through CRISPR/Cas9.^19^ First, they inserted an anti-CD19 CAR cassette containing 4-1BB and CD3ζ into the AAVS1 safe-harbor locus to demonstrate the feasibility of non-viral CAR-T-cells. Then, an innovative CAR-T-cell was developed by integrating the anti-CD19 CAR cassette into the *PDCD1* locus (**Fig. 1B**). These non-viral anti-CD19 CAR-T-cells exhibited a superior ability to eradicate cancer cells both in vitro and in xenograft models. Moreover, a phase 1 clinical trial was performed to evaluate the safety and efficacy of non-viral *PDCD1*-integrated anti-CD19 CAR-T-cells in treating patients with relapsed/refractory aggressive B-cell non-Hodgkin lymphoma. In this study, 87.5% (7 out of 8) of patients achieved complete remission after 12 months. In addition, durable responses without serious adverse events were observed in all 8 patients. Interestingly, the results of single-cell sequencing in this study show that a high proportion of memory T-cells are present in the non-viral *PDCD1* site-integrated anti-CD19 CAR-T-cell products. These analyses are consistent with the robust and long-lasting anti-tumor effect in xenografts. By analyzing the single-cell sequencing data, the superior clinical efficacy of non-viral *PDCD1*-integrated anti-CD19 CAR-T-cell products is due to the higher proportion of memory T-cells.

This inspiring study shows that non-viral site-directed integrated CAR-T-cell products elicit excellent clinical safety and anti-tumor efficacy. The feasibility of non-viral site-directed integrated T-cell therapy in clinical applications was also demonstrated. This technological innovation lays a solid foundation for the development of more site-directed modified CAR-T therapies in the future. The success of this study sheds light on the establishment of a new CAR-T-cell technology platform with better clinical outcomes and fewer toxic side effects in the treatment of refractory relapsed B-cell lymphoma. Clinical results from more patients with longer follow-up will be necessary to reveal the long-term efficacy and persistence of the non-viral *PDCD1* site-specific integration anti-CD19 CAR-T-cells. Further investigations are warranted to uncover the mechanisms underlying the formation of memory T-cells in non-viral *PDCD1* site-integrated CAR-T-cells compared with viral CAR-T-cells. It will be interesting to examine downstream pathways in T-cells that are activated post viral transduction and CRISPR−Cas9 plus DNA electroporation. In addition, a recent study revealed that PD-1 checkpoint inhibition promotes the proliferation of CD62L^+^ precursors of exhausted T-cells (Tpex) in an MYB-dependent manner and maintains long-term responsiveness to immunotherapy.^20^ It is important to explore the MYB expression and proportion of CD62L^+^Tpex in *PDCD1* site-integrated CAR-T-cells to further explain the mechanism of the higher memory T-cell proportion in *PDCD1* site-integrated CAR-T-cells. In conclusion, this study demonstrates the safety and efficacy of non-viral *PDCD1* site-integrated anti-CD19 CAR-T-cells and allows for the “killing of two birds with one stone” strategy by integrating a CAR cassette into a targeted gene locus for CAR expression and gene ablation.

In the past few years, many researchers, including our team, have attempted to improve the efficacy of CAR-T-cells against solid tumors. To achieve this goal, it is pivotal to improve the persistence and maintain the cytotoxicity of CAR-T-cells in the immunosuppressive tumor microenvironment. Recently, we demonstrated that overexpression of constitutively active GP130 in CAR-T-cells results in a more robust anti-tumor capacity, better persistence and less GVHD (graft-versus-host disease) in solid tumor-derived xenograft models.^21^ We also showed that the expression of the Toll/interleukin-1 receptor (TIR) domain of Toll-like receptor 2 (TLR2)^22^ or the DAP10 cytoplasmic domain^23^ augments the expansion and anti-tumor effects of CAR-T-cells. Similar strategies can be used to develop integrated CAR cassettes against GPC3, MSLN, and PSCA to further improve the effector functions and persistence of CAR-T-cell products for treating solid tumors.

This is the first article published by scientists from China in the field of CAR-T-cell therapy in *Nature*, reporting the first CAR-T-cell product with CAR cassette integration into the *PDCD1* locus with robust preclinical and clinical results. This study presents a paradigm for collaboration between scientists from translational and clinical research in the field of immunocellular therapy. The future of CAR-T-cell technology holds bright prospects due to the fast-paced development of gene-editing techniques and the evolving understanding of the mechanisms regulating T-cell memory and exhaustion formation.
